# Towards Comprehension of the *ABCB1*/P-Glycoprotein Role in Chronic Myeloid Leukemia

**DOI:** 10.3390/molecules23010119

**Published:** 2018-01-07

**Authors:** Raquel C. Maia, Flavia C. Vasconcelos, Paloma S. Souza, Vivian M. Rumjanek

**Affiliations:** 1 Laboratório de Hemato-Oncologia Celular e Molecular and Programa de Hemato-Oncologia Molecular, Instituto Nacional de Câncer (INCA), Praça da Cruz Vermelha, 23, 6° andar, CEP 20230-130 Rio de Janeiro, Brazil; fvasconcelos@inca.gov.br (F.C.V.); paloma.inca@gmail.com (P.S.S.); 2 Laboratório de Imunologia Tumoral, Instituto de Bioquímica Médica Leopoldo de Meis, Universidade Federal do Rio de Janeiro (UFRJ), Av. Carlos Chagas Filho, 373, Cidade Universitária, CEP 21941-902 Rio de Janeiro, Brazil; vivian@bioqmed.ufrj.br

**Keywords:** chronic myeloid leukemia, P-glycoprotein, tyrosine kinase inhibitor, drug resistance, microvesicles, inhibitor apoptosis proteins, microRNAs, new compounds

## Abstract

The introduction of imatinib (IM), a *BCR-ABL1* tyrosine kinase inhibitor (TKI), has represented a significant advance in the first-line treatment of chronic myeloid leukemia (CML). However, approximately 30% of patients need to discontinue IM due to resistance or intolerance to this drug. Both resistance and intolerance have also been observed in treatment with the second-generation TKIs—dasatinib, nilotinib, and bosutinib—and the third-generation TKI—ponatinib. The mechanisms of resistance to TKIs may be *BCR-ABL1*-dependent and/or *BCR-ABL1*-independent. Although the role of efflux pump P-glycoprotein (Pgp), codified by the *ABCB1* gene, is unquestionable in drug resistance of many neoplasms, a longstanding question exists about whether Pgp has a firm implication in TKI resistance in the clinical scenario. The goal of this review is to offer an overview of *ABCB1*/Pgp expression/activity/polymorphisms in CML. Understanding how interactions, associations, or cooperation between Pgp and other molecules—such as inhibitor apoptosis proteins, microRNAs, or microvesicles—impact IM resistance risk may be critical in evaluating the response to TKIs in CML patients. In addition, new non-TKI compounds may be necessary in order to overcome the resistance mediated by Pgp in CML.

## 1. Introduction

Chronic myeloid leukemia (CML) is a form of hematopoietic stem cell disease characterized by the presence of the oncogene *BCR-ABL*, which is created by the fusion of *BCR* and *ABL* genes and results in a constitutively active *BCR-ABL* tyrosine kinase protein [1]. This protein stimulates a number of cell survival signaling pathways, such as the PI3K/AKT/mTOR and RAS/RAF/MEK/ERK pathways, resulting in uncontrolled proliferation and apoptosis inhibition [2,3].

Imatinib mesylate (IM), described by Druker et al. [4], was the first tyrosine kinase inhibitor (TKI) approved for CML treatment in the USA by the Food and Drug Administration (FDA) in 2001 [5]. IM is widely used around the world as first-line treatment for CML patients. However, 30% to 40% of CML patients exhibit disease progression, relapse, and/or intolerance to IM [6,7]. More potent second-generation TKIs—such as dasatinib, nilotinib, bosutinib, and ponatinib [8,9,10,11]—do not overcome resistance or side effects [12,13] in all patients.

The quantification of *BCR-ABL1* transcript levels by quantitative real-time PCR (qRT-PCR) remains the most sensitive method for analyzing clinical response to TKIs. Based on the IRIS study [14], major molecular response (MMR) is defined as a ≤0.1% 3 log reduction in the *BCR-ABL1* transcript according to the international standard (IS) [15], and complete molecular response (CMR) as a reduction of 4.5 log according to the IS: <0.0032%. The term “CMR” was recently substituted by the term “molecularly undetectable leukemia” [16].

In the last 15 years, IM treatment has had a great impact on the efficacy and survival of CML patients. At a 12-month time point and with respect to long-term outcome [15], MMR rates were improved with IM as compared to the combination of interferon-alpha and cytosine arabinoside which was used prior to the IM era [17]. Castagnetti et al. [18] confirmed the prognostic value of MMR 12 months after IM treatment. MMR rates at 12 months were 49%, and six-year overall survival (OS) was 89%. Also, Hughes et al. [19] demonstrated that MMR at 12 months was predictive of a low risk of disease progression. Another work showed progression-free-survival of 82% and OS of 84% [20].

Occurrence of *BCR-ABL1* mutations is considered the most frequent cause of unfavorable clinical TKI response [6,21]. Among the mutations, the threonine-to-isoleucine substitution at residue 315 (the T315I mutation) confers a high level of resistance, not only to IM but also to dasatinib, nilotinib, and bosutinib. Only ponatinib is capable of being effective in patients with this specific mutation [12]. In this situation, treatment with ponatinib, despite the risk of a thrombotic event, should be considered [22]. In a recent work, the T315I mutation was found in approximately 16% of patients in any phase of CML [23]. Other studies found mutations in approximately 40% of patients resistant to IM in any phase of this disease [24]. In fact, many other factors are implicated in IM resistance, such as *BCR-ABL1* amplification and/or overexpression and intolerance or lack of adherence to IM [25].

Both *BCR-ABL*-dependent and -independent mechanisms of resistance have been described [26]. Among the independent mechanisms, the bone marrow environment and quiescent CML stem cells have been considered protective factors for CML cells against the effects of TKIs [27,28]. In addition, efflux membrane transporters, which are mechanisms involved in the multidrug resistance (MDR) phenotype [29] have been exhaustively studied in diverse neoplasms and are also related to CML treatment failure [30]. The efflux proteins belonging to the ATP-binding cassette (ABC) superfamily are represented by 49 genes, and some ABC members are associated with cancer and other diseases [31]. Several members of the *ABCB*, *ABCG*, *ABCA*, and *ABCC* subfamilies are related to cancer [32]. The *ABCB1*/pump P-glycoprotein (Pgp) was first described by Juliano and Ling [33] as a 170-kDa cell surface glycoprotein which showed a membrane permeability barrier ‘function’in drug-resistant cells. Currently, much is known about Pgp drug efflux function. Pgp pumps out a wide range of substrates across the cell membrane and its pump activity is closely related to MDR [30]. Studies have shown that MDR proteins, mainly Pgp, play an important role in drug efflux, including that of TKIs [34]. Additionally, some studies have demonstrated that Pgp is associated with cell survival and apoptotic pathways [35]. Notwithstanding, 40 years after the discovery of Pgp [33], there is still no solid confirmation regarding the role of *ABCB1*/Pgp in CML patients. Also, *ABCB1*/Pgp expression and activity need to be more fully evaluated with respect to TKI resistance in CML patients. As a result, we believe it is important to review the pre-clinical approaches involving *ABCB1*/Pgp expression, activity, and single nucleotide polymorphisms (SNPs) in CML to better understand the role of this protein in the MDR phenotype in the clinical setting.

## 2. Clinical Relevance of *ABCB1*/Pgp Expression and Activity in CML Patients

Studies using samples from CML patients could be very relevant, because they allow the possibility of comparing the results obtained in in vitro with clinical treatment response. Furthermore, Pgp expression and/or activity as well as *ABCB1* mRNA have been detected in CML samples from patients in studies conducted by diverse groups.

### 2.1. ABCB1/Pgp Expression/Activity in Different CML Phases/Stages

The Pgp efflux transport activity and expression have been analyzed in samples of patients at various phases of CML to understand the Pgp contribution in TKI resistance.

Fifteen years ago, Carter et al. [36] employed tetramethylrosamine (TMR), a dye used for functional assay of MDR. MDR activity was analyzed by uptake/retention of TMR with no addition of modulatory agents. They analyzed 34 samples from CML patients and 39 samples from healthy individuals. Cells from patients in the accelerate phase (AP) retained less TMR than cells from patients in the chronic phase (CP), and peripheral blood mononuclear cells (PBMC) from healthy individuals. The authors found no association between the energy-dependent efflux of TMR or Rhodamine-123 (Rho-123; another fluorochrome for MDR activity) and *ABCB1* mRNA levels. Both PBMC and CML cells exhibited variable *ABCB1* mRNA levels with no detectable difference between the samples.

In different Brazilian cohorts using CML patient samples, the fluorochrome Rho-123 was used in association with the modulator cyclosporine A (CSA) to evaluate the MDR activity by flow cytometry. In 2007, Vasconcelos and colleagues analyzed the MDR activity in 62 CML samples from 45 CP, 7 AP, and 10 blast phase (BP) patients [37]. The choice of a cut-off for positivity was based on Pgp-positive and Pgp-negative CML cell lines. The number of positive patient samples was similar among the CP, AP, and BP of CML. In 2011, the same group analyzed a larger number of samples from patients in advanced phases of CML (12 AP and 48 BP) and found similar results [38]. Additionally, another strategy to analyze the results was employed. The positive samples were divided into high or low MDR activity using arbitrary cut-off. This time, the majority of samples with high MDR activity were in the BP; this is in accordance with Carter’s results [36]. Even when samples were analyzed without an established cut-off, the advanced stages of CML exhibited higher levels of MDR activity, as described in Vasconcelos et al. in 2013 [39]. Samples from 13 CML patients in advanced phases exhibited higher levels of MDR activity when compared to those of the 42 CP patients (*p* = 0.0318) [39]. One possible explanation for the higher MDR activity could be prior treatment, including IM, in the BP group, since it is well known that diverse drugs may induce Pgp expression/activity. Another important question is to determine if Pgp protein expression could be associated with IM resistance, independently of its activity. Cells from untreated 55 CP CML patients had higher Pgp protein levels (detected with monoclonal antibody by flow cytometry) than 13 patient cells of advanced phases (*p* = 0.0022) [39]. Additionally, Pgp expression levels were not associated with MDR activity independent of the method applied [37,38,39]. This raises the question as to whether the dissociation observed between Pgp activity and Pgp expression could be influenced by polymorphisms, as was previously observed by Vivona et al. [40].

The differences observed in different articles could be explained by the methodology chosen to analyze the results (Table 1). Besides this, it is important to take into account the complexity of different factors which may interfere with *ABCB1*/Pgp expression/activity levels. These factors may be microRNAs or polymorphisms, which will be mentioned later.

### 2.2. ABCB1/Pgp and CML Patient Response to TKI

By analyzing Pgp activity in CML samples from different subgroups (treatment failure and treatment response to IM), Park et al. [41] revealed no differences among subgroups, or between PBMCs (from healthy individuals) and the patient groups (*p* = 0.769). However, they found significantly higher Pgp expression in the subgroup showing IM failure (*p* = 0.031).

In another study, Vasconcelos et al. [39] analyzed 55 CML samples in vitro. After IM incubation, they defined resistant or sensitive CML samples regarding to *BCR-ABL* protein activity inhibition by IM. The authors also observed lower Pgp activity in IM-sensitive than IM-resistant samples (*p* = 0.0255) and in healthy PBMC (*p* = 0.0034). However, no difference was observed between IM-resistant and PBMC samples (*p* = 0.9109). The analysis of Pgp protein expression (using monoclonal antibody by flow cytometry) was not predictive of resistance or sensitivity in CML samples incubated in vitro with IM. The IM-resistant and -sensitive patient samples showed the same levels of Pgp expression (*p* = 0.2413) [39]. In conclusion, this work demonstrated that Pgp expression levels were not associated with in vitro IM resistance.

Another question is related to the impact of Pgp protein expression in CML patients after receiving treatment. Studies have shown that Pgp expression might arise spontaneously or might be induced by drugs [48]. Our group analyzed Pgp expression in 47 patient samples in different time points of disease progression. The results showed that independent of CML phase, Pgp expression levels were increased during progression of disease. Additionally, in vitro data showed that IM induced an increase of Pgp protein and mRNA levels in MDR CML cell line. Together, these data support that IM might contribute to MDR phenotype in CML [38].

Besides Pgp expression/activity, many authors analyzed *ABCB1* mRNA levels to observe some relationship with clinical response to TKIs. Razga et al. [43] investigated the predictive value of *ABCB1* mRNA levels in samples from 30 CML patients with respect to the response to IM treatment. They did not find a statistically significant relationship between *ABCB1* mRNA levels and IM response at 6 and 12 months. In line with these findings, Malhotra et al. [44] observed that 44 out of 63 CML patients achieved CMR or MMR, by *BCR-ABL* quantification. Both CMR and MMR patients were included in the responder category. The *ABCB1* expression levels were analyzed and there was no difference between responder or non-responder patients. However, our group observed low *ABCB1* mRNA levels in patients that achieved MMR at 12 months from the start of IM treatment [45]. In contrast, non-responder patients had the highest *ABCB1* levels. There was no difference with respect to eight-year OS in responder and non-responder patients, suggesting that other factors are important for long-term OS.

Agrawal et al. [46] verified high *ABCB1* gene expression levels after 24 months of IM treatment. There was a significant association between these high *ABCB1* levels and MMR. Interestingly, patients presenting high *ABCB1* levels, analyzed at the time of IM resistance, demonstrated a response to nilotinib given as a second-line treatment.

Eaddie et al. [47] analyzed 155 CML patients at day 22 post-IM therapy, and observed that samples with high *ABCB1* mRNA levels showed undesirable responses compared to samples with low *ABCB1* mRNA levels. The increase of *ABCB1* was predictive of reduced early molecular response at 3 months and reduced MMR at 12 months. In contrast to Agrawal et al. [46], Eaddie et al. [47] demonstrated that a group of IM non-responder patients presenting high *ABCB1* mRNA also failed to respond to subsequent treatment with nilotinib using the Tidell II protocol [49]. This study indicates that early monitoring using *ABCB1* mRNA levels may represent an accessible marker for predicting early response or resistance to IM and nilotinib, which are both Pgp substrates [50,51].

The different methodologies for Pgp analysis may explain the divergence among the authors in explain their results. In addition, the different analysis of protein, mRNA, and activity of Pgp may contribute tothis complexity. Besides, Pgp alterations, such as polymorphisms and epigenetic changes will be discussed below.

## 3. *ABCB1* Polymorphisms (SNPs) in Chronic Myeloid Leukemia Patients

Pgp belongs to the ATP binding cassette (ABC) family and is encoded by the *ABCB1* gene located in the 7q21.1 chromosome. The highly polymorphic *ABCB1* gene has 28 exons that encode a 170-KDa transmembrane protein. Fifty SNPs have been described in the coding region [52]. The most common SNPs are in exon 12 (1236C > T), exon 26 (3435C > T), and exon 21 (2677G > T/A) [53]. These three variants are reported to be in linkage disequilibrium [54,55]. The impact of *ABCB1* SNPs on pharmacokinetics has not been elucidated yet. Since IM interacts with Pgp as a substrate or modulator [56], SNPs might have an influence in the IM response of CML cells. However, there is no consensus about the role of this and other SNPs in the clinical setting, as discussed below.

### 3.1. SNPs at Position 1236

The first study to demonstrate the association between *ABCB1* SNPs and response to standard dose of IM in CML patient samples was published in 2008 by Dulucq et al. [54]. They analyzed 90 patient samples from a French population in CP or AP, after receiving 400 mg of IM (standard dose) daily for 12 months. They described that most patients who achieved MMR were homozygous for the 1236T allele. However, in a posterior study the same authors reported contradictory results. They analyzed the same SNPs in a larger multicenter study with 557 patients. At this time, the 1236T allele had no impact on the achievement of MMR [57]. The same picture was observed in a study of 118 Brazillians with CP CML by Vivona et al. [53]. On analyzing samples from 52 Asian CP CML patients, Ni et al. [58] found that the 1236T allele was associated with resistance to IM. In a study of 46 Dutch CP CML patients, the C allele at the same position (1236) was associated with MMR in patients using high doses of IM [59]. On the other hand, on analyzing 215 Malaysian CML patients, it was found that homozygous 1236C was significantly associated with the occurrence of resistance [60].

### 3.2. SNPs at Position 3435

The homozygous T allele at position 3435 in a population of 46 Dutch CP CML patients reduced the probability of MMR [59]. Similar data was observed in 52 Chinese CP CML patients harboring the 3435TT/CT genotype. They showed a higher resistance rate than those with the CC genotype [58]. In contrast, some (not statistically significant) data suggested higher frequencies of MMR related to 3435TT in a Nigerian cohort with 110 CP CML patients [61]. More contradictory observations were found with genotype 3435CC. In 65 Caucasian CP CML patients, the homozygous C allele was predictive of primary failure to IM [62] while in a mixed population of 189 patients (156 Caucasians, 23 Asians, and 10 non-Asians and non-whites) there was a high rate of CMR [63].

### 3.3. SNPs at Position 2677

The relevance of the 2677 genotype has been addressed in different populations of CML patients. The association with IM response has been found in some studies. Ni et al. [58] showed that achievement of complete cytogenetic response (CCR) was associated with the 2677A allele in 52 Asian CP CML patients. The allele 2677TT was commonly associated with response to IM. However, the data are contradictory. In an Egyptian population of 100 patients in the CP of CML this allele was associated with a slightly but not significantly higher optimal response [64]. In another Egyptian study, the homozygous 2677T was associated with better molecular response in a population of 66 CP, 18 AP, and 12 BP patient samples [65]. The presence of the T allele protected Caucasian patients from primary failure to IM (total sample number 65 CML patients in CP) [62]. However, adverse effects of the homozygous T allele in positions 2677 and 3435 were associated with a lower probability of achieving MMR and CMR in a cohort of 46 Dutch CP CML patients treated with high doses of IM [59]. Regarding the 2677GT allele, it was an independent risk factor for failure in Egyptian patients in different phases of CML [65]. In a cohort of 215 Malaysian CML patients, a better CCR and a significantly lower molecular response were observed for patients with 2677G > T/A SNPs [60]. Interestingly, the 2677G allele was associated with MMR in patients that received IM 400 mg and cytosine arabinoside in a total cohort of 557 patients [57]. However, the absence of association between *ABCB1* SNPs and response to IM was found in a different population [66,67,68].

### 3.4. Haplotypes

Vivona et al. [40] divided 28 CP CML patients treated with standard doses of IM into two groups according to the presence of wild-type or mutated haplotypes. The wild-type alleles (1236C/3435C/2677G) had higher Pgp activity and failed to achieve MMR. These data may explain the findings described before by Vasconcelos et al. [39] and Park et al. [41]. These authors had also shown the similarity between Pgp activity levels in CML cells and healthy individual cells as being indicative of poorer IM response. Different SNPs cause different impacts on Pgp activity or expression. Both 1236C > T and 3435C > T are synonymous SNPs, while in 2677G > T/Aa substitution of alanine by serine (2677T) or threonine (2677A) occurs [69]. Therefore, the 1236T > C and 3435C > T SNPs should not interfere with Pgp activity. However, the SNP 3435C > T was associated with reduced *ABCB1* expression, leading to reduced Pgp activity [52,70,71]. Wang et al. demonstrated the influence of this SNP on *ABCB1* mRNA stability. When cells were transfected with this SNP, there were significantly lower transcripts in the 3435T homozygotes as compared to the 3435C homozygotes [55].

### 3.5. Susceptibility to the Development of Leukemia and ABCB1 SNPs

Susceptibility to the development of leukemia has been associated with the 3435T and 2677TT alleles, as observed by Penna et al. [72], although no association was found by Goreva et al. [73] or Jamroziak et al. [74]. Regarding CML, Yaya et al. [75], studied the genotypes 1236C > T, 2677G > T and 3435C > T in 89 patient samples using 99 healthy individual samples as controls. They found that some *ABCB1* genotypes had an influence on the susceptibility to CML. The 1236TT genotype was associated with susceptibility, while 3435CT was associated with reduced risk and the 2677GT had a protective effect on susceptibility to CML. The analysis of the combination of haplotypes showed no effect on the susceptibility to CML, but 1236CT/3435CC and 1236CC/2677GT showed a protective effect [75]. Contradictory results were obtained by Vivona et al. [53] who found no association between C1236T, C3435T, and G2677T/A SNPs and the risk of developing CML.

### 3.6. Impact of ABCB1 SNPs on Pharmacokinetics

As mentioned before, some patients interrupt TKI treatment because of poor tolerability or treatment failure [6,7]. The impact of *ABCB1* SNPs on pharmacokinetics has not been elucidated yet. Gurney et al. reported that the presence of a homozygous genotype for the T nucleotide at 1236C > T, 2677G > T/A, and 3435C > T showed higher steady-state IM clearance when compared to the CC or GG genotypes, and required fewer dose reductions [76]. Dulucq et al. have shown that higher concentrations of IM correlated with the homozygous 1236T allele and MMR after 12 months of treatment with IM [54]. In a Nigerian population, Adeagbo et al. [61] reported that the C3435T genotype was associated with higher concentrations of IM. In a Caucasian population, Galeotti et al. [77] found the same SNP as being predictive of IM efficacy and toxicity.

Together, the differences observed between the frequencies of the alleles and the limited number of samples in some studies can explain the contradictory findings in the literature.

## 4. Interactions between *ABCB1*/Pgp and TKIs in CML Cells

The MDR phenotype, via Pgp overexpression, can intrinsically occur in cancer cells or it can be acquired over the course of chemotherapy [78]. Pgp and, consequently, MDR, can also be transferred through cellular microvesicles, but this will be discussed further later [79,80]. Regarding drug-induced Pgp expression, cell lines represent biologically relevant models to study MDR. Basically, the parental cell line is exposed to increasing drug concentrations and the small survival population may represent the resistant population [81].

In CML cell lines, extensive studies have shown upregulation of Pgp in the presence of chemotherapeutic drugs with different structures and mechanisms of action. Our group developed two resistant CML cell lines derived from K562. The K562-Lucena 1 (refered as Lucena) cell line was established using vincristine, and the FEPS cell line was generated using daunorubicin [82,83]. These cell lines are also highly resistant to IM [83]. While Pgp overexpression is the main mechanism studied in acquired drug resistance, many other changes in the gene profile can also contribute to MDR. Specifically, Moreira et al. [84] performed a microarray analysis using the K562, Lucena, and FEPS cell lines. The results showed a total of 130 genes differentially expressed between K562 vs. Lucena, and a total of 932 genes differentially expressed between K562 vs. FEPS. The gene ontology analysis showed that these MDR cell lines display genes related to cell cycle, cell death, cell morphology, cellular development, cellular growth, and proliferation. Usually, resistant K562 cell lines show a large spectrum of drug resistance, and several studies have focused on an IM CML drug-resistant cell line. The first association of IM with Pgp was described by Mahon et al. [85]. They established different CML cell lines resistant to IM. However, the authors observed Pgp overexpression in only one IM-resistant cell line. The induction of Pgp expression and the modulation of its resistance by verapamil (inhibitor of Pgp efflux activity) characterized IM as a substrate for Pgp.

Alves et al. [86] created an in vitro model which mimics the poor adherence to IM observed in patients. For these purposes, the authors established and characterized two IM-resistant cell lines derived from K562 parental cells. The K562-RC was generated by continuous exposure of IM, while K562-RD was developed based on discontinuous exposure to IM with cycles of 10 days. Both resistant cell lines showed similar levels of Pgp, and its inhibition induced a decrease of IM IC_50_. Furthermore, only the K562-RD cells presented overexpression of *BCR-ABL*. The results obtained in the K562-RD cell line may mimic the consequence of IM treatment interruption in patients.

Some reports have shown that expression of Pgp was associated with resistance to other TKIs like nilotinib [87], dasatinib [88], and bosutinib [89]. Additionally, Peng et al. [90] established an IM-resistant cell line derived from K562 cells, which exhibited *ABCB1*/Pgp overexpression and cross-resistance to nilotinib, dasatinib, and bosutinib. Moreover, there is some evidence that IM, nilotinib, and dasatinib are Pgp-substrates or modulators depending on TKI concentration. In a narrow concentration range TKIs are transported, while in high concentrations they inhibit the Pgp activity [51,91,92].

Studies have also demonstrated Pgp expression induced by drugs in the clinical scenario. Hu et al. [93] demonstrated that chemotherapeutic drugs (idarubicin, mitroxantrone, and epirubicin) induced Pgp expression in CML blasts treated ex vivo. The blast samples had a Pgp-negative status and after 16 h of drugs treatment showed upregulation of Pgp expression. Likewise, the Pgp status was analyzed in CML patients 1.3 and 5 months after chemotherapy, and the results showed Pgp-positive status. Stromskaya et al. [48] also observed that patients in AP acquired increased Pgp activity during IM therapy and development of IM resistance. In addition, they observed short survival in patients with Pgp activity while patients with no Pgp activity achieved CCR. A study developed by our group [38], showed that IM treatment induced an increase in Pgp protein and mRNA levels in Lucena cells (Pgp-positive cell line) whereas no difference was observed in K562 parental cell line. The in vitro findings could explain the observation that Pgp expression varies in patients analyzed at different phases of treatment. Taken together, these data suggest a role for Pgp in TKI resistance that could be translated to treatment failure in a clinical setting. The study of interactions between TKIs and ABC transporters is important, especially in the field of development of new drugs able to overcome, or even inhibit, the interference of efflux transporters in the pharmacokinetics of drugs.

## 5. Resistance Mediated by Pgp Associated with Cellular Microvesicles (MVs)

As previously discussed, chemotherapeutic drugs can induce *ABCB1*/Pgp expression [94]. However, other mechanisms have been investigated with respect to MDR phenotype development. Bebawy et al. [79] first demonstrated that Pgp can be transferred from drug-resistant cancer cells to drug-sensitive cells via membrane microparticles (MPs). MPs are small enclosed vesicles derived from the cell membrane [95]. In the work by Bebawy, it was shown that MDR leukemia cells shed MPs carrying Pgp and that MPs did transfer a functional protein, and consequently the MDR phenotype, to drug-sensitive cells. Later, Bebawy’s group also demonstrated that *ABCB1* mRNA and the *ABCC1*/MRP1 protein and mRNA could also be carried by MPs derived from MDR cells [96,97]. Our group also demonstrated that Pgp-positive CML cells can spontaneously shed MPs carrying Pgp, mRNAs, and microRNAs (miRNAs). In addition, our group described for the first time how MPs derived from CML cells carry inhibitor apoptosis proteins (IAPs), mRNAs, and proteins which contribute to a multifactorial resistance phenotype [80]. In another perspective, Lopes-Rodrigues et al. [98] reported that drug-resistant CML cells shed larger microvesicles (MVs) than drug-sensitive CML cells, and present a protein content associated with MDR biomarkers. Recently, Milani et al. [99] analyzed RNA cargo in MVs derived from CML cell lines and identified *BCR-ABL* and other translocation signatures. In addition, in K562-MVs they identified genes related to cell communication, cell migration, and signaling pathways, etc. Common genes between K562-MVs and K562 cells were related to hematological disease, hematopoiesis, and downstream CML pathway *BCR-ABL*. MVs contained oncogenic hallmarks from the parental donor K562 cell line and were also able to transfer their cargo and induce proliferation in normal human bone marrow-derived mesenchymal stem cells.

Since MVs originate from MDR cancer cells, they are effective disseminators of the drug-resistance phenotype. Corrado et al. [100] showed that CML cells release nanovesicles (exosomes) loaded with interleukin 8 (IL-8) which promotes the proliferation and survival of leukemia cells in vitro and in vivo, using a xenograft CML tumor model. In another study, it was demonstrated that mice with CML treated with exosomes derived from CML cells had stimulated tumor growth. In addition, mice with CML treated with CML exosomes also showed a decrease in pro-apoptotic protein levels as well as an increase in anti-apoptotic proteins levels. The authors suggest that survival and proliferation mediated through CML exosomes can be activated via ERK, Akt, and NFκB pathways [101]. Analysis in the exosomes isolated from the blood samples of 13 newly diagnosed CML patients showed the presence of molecules that activate the oncogenic pathway associated with aggressiveness and the chemoresistance phenotype [102].

Additionally, detection of MVs in different leukemia subtypes is useful for diagnosis. Usually, cells from CML patients release higher amounts of MVs than those of healthy individuals, and subsequently the MV cargo reflects upon the donor cells [103].

## 6. Associations between Pgp and ‘Onco-Molecules’: Exploring Multifactorial Resistance 

Since drug resistance is probably a multifactorial phenomenon, it is important to verify associations and interactions or simultaneous overexpression of the MDR proteins, and their possible consequences with respect to CML cells.

### 6.1. Pgp and microRNAs (miRNAs) Interactions in CML Cells

Studies have shown that epigenetic modifications may regulate *ABCB1*/Pgp, as miRNAs are differentially expressed in tumor-resistant cells [104,105]. MiRNAs are small noncoding RNA molecules which regulate gene expression in a post-transcriptional manner [106]. The importance of miRNAs with respect to the regulation of Pgp and other ABC transporters was extensively discussed by Haenisch et al. [107]. MiRNAs may regulate the expression of Pgp in solid tumors and leukemia [108,109] by direct interaction in the promoter region and 3’-UTR sequence of *ABCB1*. Alternatively, miRNAs may target other mRNAs and then modify the expression levels of proteins that modulate Pgp expression [110].

Summarizing, the regulation of Pgp expression by miRNAs is very complex and studies are controversial. With respect to miR-27a and miR-451, studies have shown that both may be associated with upregulation or downregulation of Pgp expression [107].

Recently, our group described the expression of miR-27a and miR-451 in the CML Pgp-positive cell line Lucena. In addition, Lucena cells could release both miRNAs in MPs and transfer them to non-Pgp cell lines. The recipient cells acquired Pgp expression and the MDR phenotype [80]. Conversely, Feng et al. [111] demonstrated that Pgp was overexpressed in three doxorubicin-resistant CML cell lines as compared to parental K562 cells. MiR-27a expression levels were the highest in K562 cells and decreased in the resistant cells. The authors showed that *ABCB1* expression levels were directly regulated by miR-27a in K562 parental cells and also inversely correlated with *ABCB1* expression. Besides, transfection of exogenous miR-27a induced *ABCB1* downregulation in K562 Pgp-positive cells, leading to an increase in doxorrubicin sensitivity [111].

Studies have shown that miRNAs are also associated with IM response. Li et al. [105] demonstrated that miR-29b, a member of miR-29 family, acts as a tumor suppressor in K562 CML cells by suppressing proliferation and inducing apoptosis. Another study showed that miR-181a overexpression inhibited CML cell growth and increased the IM sensitivity [112]. Mosakhani et al. [113] observed that another member of miR181 family, named miR181C, was downregulated in IM-resistant cells. Li et al. [105] verified that miR-203 overexpression increased the sensitivity to IM treatment in IM-resistant cells presenting the T315I mutation. This is an interesting finding, since the T315I mutation is a big challenge in CML treatment. MiR-150 is also related with CML. It was found that miR-150 has a role as a diagnostic biomarker [114] and, despite the paucity of patients, it is also associated with IM response in CML [115].

Li et al. [116] demonstrated that miR-9 was downregulated in CML-resistant K562 cells in comparison to sensitive cells. The authors showed that *ABCB1* is a direct target of miR-9 in CML. miR-9 expression was also analyzed in 61 samples from CML patients. They found Pgp positivity in 33 (54.1%) patients, with lower miR-9 expression compared to the Pgp-negative group of patients [116]. Therefore, miR-9 could be a promising target for therapeutic interventions.

The miRNA expression profile has been widely analyzed to identify biomarkers or gene targets for therapy. Recently, Ohyashiki et al. [117] analyzed circulating miRNAs in CML patient plasma and miRNAs in exosomes derived from CML patients using the TaqMan low-density array. Initially, the authors identified 69 miRNAs differentially expressed in plasma from CML patients who had discontinued IM, as compared to healthy volunteers. Levels of miR-215 and miR-134 were found to be statistically significant. However, only downregulation of miR-215 reflected exosomal miRNA expression. In addition, low levels of miR-215 were associated with higher IM uptake. In silico analysis showed that miR-215 is related to the cellular metabolic process, cell cycle, DNA repair, and cellular stress. Yap et al. [118] performed miRNA sequencing analysis for CML patients resistant to IM. The authors identified 43 miRNAs as downregulated and 11 miRNAs as upregulated, as compared to two healthy volunteers. They highlighted miR-146a-5p, miR-99b-5p, and miR-151a-5p, and showed through in silico analysis that the target genes of these miRNAs are related to the Fanconi anemia/BRCA pathway.

### 6.2. Associations between Pgp and the Inhibitor of Apoptosis Proteins (IAPs)

IAPs have been associated with resistance in CML [119]. Interaction between Pgp and IAPs was also investigated by our group [120,121]. Bernardo et al. [122] demonstrated that survivin was detected in both cytoplasmic and nuclear localization of sensitive and resistant CML cell lines. After treatment with IM, sensitive K562 cells mainly displayed survivin in the nucleus and Lucena cells (Pgp positive) in the cytoplasm. The survivin modulation to the nucleus was associated with its reduced expression in the cytoplasm. Conversely, Lucena cells exhibited survivin in the cytoplasm and the rate of apoptosis induced by IM was low. These findings corroborate with studies reporting that cytoplasmic survivin is related to an unfavorable prognosis and suggest a relationship between survivin and Pgp [123]. In another study, Reis et al. [120] analyzed a group of 50 CML patients in an effort to detect an association between survivin and Pgp. Patients were categorized according to the Sokal score system to obtain prognostic information [124]. There was a significant positive correlation between survivin and Pgp expression in the late phase of CML, but not in the early phase. Besides, the highest levels of survivin were found in the groups with high and intermediate Sokal scores, as compared to the low Sokal score group of patients. Together, these results suggest that the association between Pgp and survivin may have a role in the evolution from CP to BP in CML. The association between Pgp and survivin was also investigated by de Souza et al. [121]. The authors treated K562 cells with low doses of vincristine for up to 24 h and they observed prominent cell cycle arrest followed by a progressive increase of survivin protein levels. *ABCB1*/Pgp expression levels were also increasingly induced by vincristine treatment of K562 cells. Concomitantly, the authors observed an increase of survivin in the cytoplasmic compartment. The simultaneous Pgp and survivin enhancement after vincristine treatment suggests a similar regulatory pathway for drug resistance in CML cells. This work is also in agreement with that of Bernardo et al. [122] regarding cytoplasmic survivin in CML-resistant cells.

In another study, Pgp was associated with another IAP, the XIAP protein [42]. The authors analyzed the expression of both XIAP and Pgp in 48 samples from CML patients. A significant positive correlation was observed between these proteins (*p* = 0.026). Also, in vitro data showed that Lucena cells (Pgp positive) were more resistant to IM treatment than K562 cells. In Lucena cells, but not K562 cells, IM caused XIAP upregulation. The authors showed that even after XIAP knockdown, Lucena cells remained more resistant than K562 cells. Our results are in line with those found in the studies of Seca et al. [125], which showed that downregulation of XIAP and Pgp is necessary to overcome IM resistance in Pgp-overexpressing cells.

## 7. Overcoming Drug Resistance in CML

Although many patients with CML achieve CMR after IM therapy, a sub-group of patients fails to respond or show treatment resistance. In this situation a strategy may be necessary, and we will discuss this bellow.

### 7.1. Clinical Modulation of Pgp

Concerning clinical Pgp modulation, few studies have been performed with CML patients. List et al. [126] conducted a randomized study comparing patients treated with daunorubicin plus high-dose cytarabine and patients treated with the same protocol containing cyclosporine (CSA). These authors verified that Pgp expression adversely influenced complete remission or restored CP. However, they also demonstrated that clinical modulation of Pgp activity with chemotherapeutic drugs plus CSA showed no benefit in terms of contributing to a complete remission rate or returning patients to the CP.

Twenty years ago, our group treated 15 heavily pretreated patients presenting diverse types of leukemia with etoposide and CSA in an effort to bypass MDR mediated by Pgp [127]. Among these patients, six presented CML in the BP. In that group, only one patient obtained a good response, which was characterized by a return to the CP and over one year of survival. The other five CML patients presented minor response or failure. This type of Pgp modulation was removed from consideration as a clinical approach due to its high toxicity.

### 7.2. Interaction of Second- and Third-Generation TKIs with Pgp

Using CML cell lines, many authors [128,129,130], including those of our group, have demonstrated that IM resistance may be due to Pgp. In fact, besides the importance of amplification or the mainly point mutations of the *BCR-ABL* gene, one of the most investigated resistant mechanisms in CML is that conferred by the efflux pump, Pgp. This efflux can be reversed by inhibiting Pgp transport activity. In this context, besides its role as substrate of Pgp, IM can be a weak modulator agent [42,56].

In an interesting study about the interactions between TKIs and ABC transporters, Dohse et al., using in vivo and in vitro studies [92], demonstrated that the second-generation TKI nilotinib interacted less intensely with Pgp and *ABCG2* when compared to IM. However, it was a more potent inhibitor of both efflux pumps. Also, Tiwari et al. [87] verified that nilotinib efficiently inhibited *ABCB1* and *ABCG2* efflux activity. These findings are in accordance with the favorable molecular response (*BCR-ABL* load before clinical nilotinib treatment) observed in patients treated with nilotinib after IM resistance, exhibiting high *ABCB1* gene expression [46]. Furthermore, it is well known that *ABCB1* and *ABCG2* are expressed in a drug-resistant subpopulation of leukemia stem cells (CD34+ CD38−) [131,132]. In accordance, Wang et al. [133] showed that nilotinib targeted CD34+ CD38− cells and increased the efficacy of doxorubicin and mitoxantrone by blocking the efflux activity of both *ABCB1* and *ABCG2*. Taken together, these data suggest that nilotinib may be of particular relevance in IM-resistant patients expressing *ABCB1*/Pgp.

With respect to the second-generation TKI dasatinib, using an *ABCB1* overexpressing cell line, Hiwase et al. [88] demonstrated that this TKI—in addition to being more potent than IM—is also transported by *ABCB1*/Pgp, and is therefore a substrate for this efflux pump. In addition, the authors verified that PSC833, an *ABCB1* inhibitor, increases the intracellular uptake and retention of dasatinib.

To investigate the functional consequences of *ABCB1* involved in bosutinib (also a second-generation TKI), Redaelli et al. [89] used in vitro and in vivo experiments. First, they used IUR assay and observed that high *ABCB1* levels corresponded to decreased intracellular C-14 radiolabeled bosutinib. Next, they used an in vivo xenograft model to investigate the anti-tumor activity of bosutinib. Nude mice injected with a CML cell line overexpressing *ABCB1* and treated with bosutinib exhibited less of a response compared to nude mice injected with cells silenced for *ABCB1*.

With respect to ponatinib, it was first demonstrated by Sen et al. [134] that this third-generation TKI is able to inhibit both *ABCB1* and *ABCG2* efflux pumps. Next, Lu et al. [135] demonstrated that ponatinib is not transported from cells via Pgp, *ABCG2*, and OCT1. In relation to Pgp, using the CML K562 cell line and its *ABCB1* overexpressing variant K562-dox in the presence of cyclosporine and pantoprazole (both Pgp inhibitors), there was no difference in the intracellular uptake and retention of ponatinib between the two cell lines. This result indicates that ponatinib is not extruded by Pgp [135]. However, ponatinib may be associated with an increased risk of serious thromboembolic events, which was verified in a phase II trial by Cortes et al. [10].

### 7.3. Efficacy of Non-TKI Drugs in CML

In vitro studies have suggested curcumin, a phytochemical which has been widely used due to its diverse pharmacological activities as an antibacterial [136] and anti-cancer agent [137], in an attractive strategy for overcoming MDR. Recently, it was shown by Lopes-Rodrigues et al. [138] that not only curcumin, but also curcumin derivatives are inhibitors of Pgp. Curcumin and a curcumin derivative named curcumin 10 inhibited Pgp activity and expression in a CML cell line overexpressing Pgp (K562Dox). Other authors [139] also found a relevant inhibitory effect of the activity and expression of Pgp in CML cells treated with diketone and cyclohexanone curcumin analogs. Zhang et al. [140] demonstrated that Euphorbia factor L1, a diterpenoid isolated from *Euphorbia lathyris*, was able to inhibit the efflux activity of Pgp. In addition, this reversor agent enhanced the intracellular accumulation of doxorubicin and vincristine in the CML K562/ADR cell line overexpressing Pgp [140].

Taking into account that drug resistance is probably a multifactorial phenomenon, it is important to verify the association, interaction, or simultaneous overexpression of the MDR proteins and their possible consequences in CML cells. In this situation, a new treatment strategy may be necessary.

Based on the association between Pgp and IAPs, an important approach could be the treatment of patients with drugs that can simultaneously inhibit proteins involved with drug resistance. In this way, our group has been investigating a new drug named LQB-118, a pterocarpanquinone structurally related to lapachol, which has previously shown efficacy against leukemic cell lines [141]. We demonstrated in K562 cells and Lucena cells (Pgp positive), that LQB-118 was similarly effective in reducing viability, as determined by MTT assay. LQB-118 was also capable of inducing apoptosis in both cell lines. The apoptotic rate increased when cells were exposed to LQB-118 for a longer period of time (72 h) [142]. In parallel, we analyzed CML cells from 13 patients to observe the capacity of LQB-118 to induce apoptosis. After 48 h incubation, LQB-118 induced a median of 60.18% apoptosis (range: 40 to 85%). The LQB-118 compound was able to induce a high apoptosis rate in CML cells from patients not only overexpressing active Pgp, but also exhibiting p53 and MRP1 overexpression [142]. These data evoke questions which help us to understand the need to reach multiple targets for better treatment results in CML patients. In this context, we also verified that LQB-118 was capable of inhibiting CML cells from overexpressing IAPs. When CML cell lines were treated with LQB-118, survivin and XIAP were downregulated at a higher level in the resistant Lucena cell line compared to the Pgp-negative K562 cell line. These findings suggest that LQB-118 is a potent compound with potential to target multifactorial resistant mechanisms.

Different pathways have been proposed to explain the mechanisms of action of LQB-118 in CML. de Sá Bacelar et al. [143] investigated cell death induction by LQB-118 in a K562 cell line. In agreement with Maia et al. [142], the authors confirmed the cytotoxic effect of LQB-118 on apoptosis induction. They also reported that LQB-118 quickly led to the increase of intracellular calcium levels and it was dependent on the extracellular microenvironment. Moreover, this compound induced an increase in reactive oxygen species (ROS) through its reduction at the mitochondria. Finally, the authors showed that LQB-118 induced time- and dose-dependent endoplasmic reticulum stress (ER) via the caspase-12 activation pathway [143]. The LQB-118 mechanism of action in CML cells was further studied by de Faria et al. [144]. Their findings showed a reduction of a nuclear fraction of NFκB after drug treatment, with no changes in Akt or MAPK upstream pathways. However, LQB-118 could partially inhibit proteasome activity in CML cells and also modulate miR-9 and miR-21. Both miRs were found downregulated in K562-sensitive cell line with no changes in NFκB1 protein levels after LQB-118. In contrast, miR-9 and miR-21 were found to be upregulated in Pgp-positive cells, with a subsequent reduction of NFκB1 levels, which is probably associated with NFκB pathway inactivation after drug treatment. An understanding of the mechanisms of LQB-118 would be helpful for overcoming TKI resistance in CML. These findings suggest LQB-118 as a potent compound with the potential to target multifactorial resistant mechanisms.

## 8. Conclusions and Future Prospects

Since the discovery of Pgp in the 1970s, many authors have reported its association with drug response in clinical practice. For many cancer types, elevated expression levels of Pgp affect the efficacy of cancer therapy. CML has been discussed in this respect. For this reason, in the present review we explored the genuine role of Pgp in CML since different cohorts of patients exhibit *BCR-ABL1*-independent TKI resistance.

**Figure 1:**
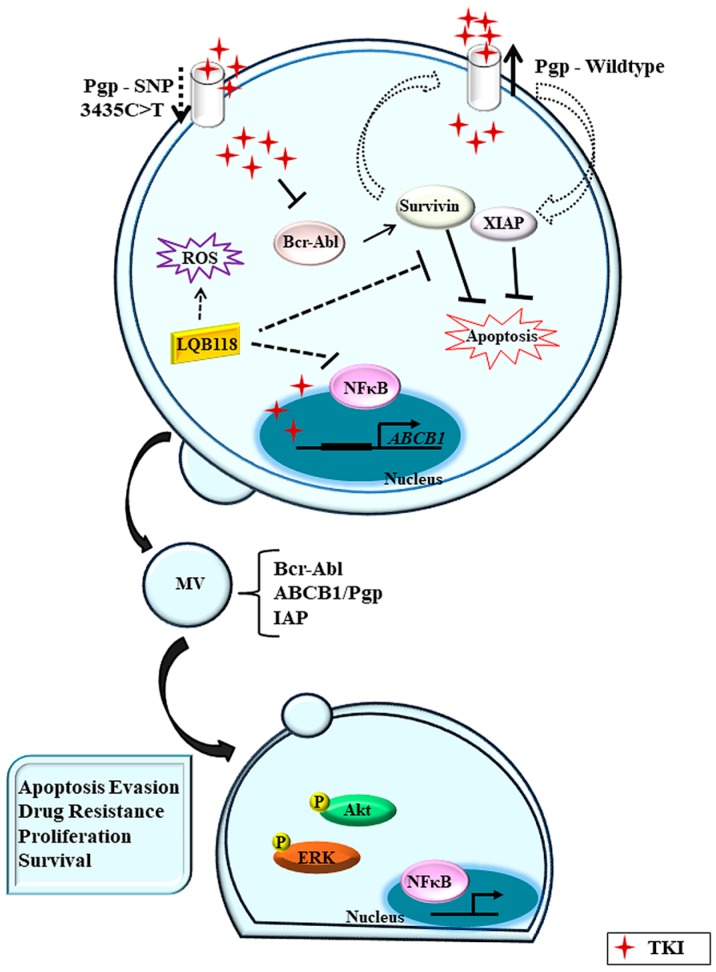
Multidrug resistance in chronic myeloid leukemia. P-glycoprotein (Pgp) expression in chronic myeloid leukemia (CML) cells promotes tyrosine kinase inhibitors (TKI) efflux and a consequent reduction of intracellular drug concentration. On the other hand, some single nucleotide polymorphisms (SNP) may reduce Pgp drug efflux activity and then TKIs can inhibit Bcr-Abl protein. However, high intracellular drug concentration may induce Pgp mRNA transcription. Pgp may also contribute to apoptosis inhibition through IAP proteins (survivin and XIAP). Nevertheless, the new compound LQB-118 could overcome MDR through ROS induction, NFκB pathway inhibition, and IAP downregulation. Anyway CML cells release cellular microvesicles (MV) carrying oncogenic molecules whose cargo can be transferred to microenvironment cells.

In vitro data support that IM and other TKIs have an affinity to Pgp and may act as its substrates or function inhibitors depending on drug concentration [51,92]. Nevertheless, the translational and clinical data are variable, and the role played by the efflux activity of Pgp in resistance is not so clear. Undoubtedly, IM and all subsequent generations of TKI represent a great advance in the treatment of CML. However, other *BCR-ABL1*-independent mechanisms may contribute to unfavorable scenarios, and the major challenge remains how to overcome drug resistance (Figure 1).

As discussed above, diverse studies support the contribution of Pgp, with or without interaction with other molecules, to resistance to TKIs in CML. Ongoing studies probably will unravel the role of Pgp in CML resistance in order to improve the therapeutic approach clinically. In keeping with studies demonstrating the ineffectiveness of TKIs in some CML patients, new drugs should be investigated, with or without a combination with TKIs. The new therapeutic agents should ideally be capable of treating *BCR-ABL*-independent mechanisms of resistance, such as Pgp overexpression, besides showing significant activity in CML cells.

## Figures and Tables

**Table 1:** *ABCB1*/Pgp/expression/activity in samples from chronic myeloid leukemia patients.

Method	Number of Samples	Clinical Relevance	Reference
Tetramethylrosamine assay	34	Samples from patients with newly diagnosed CML showed resistance in comparison with cells from healthy donors. In later CML phases, samples exhibited more resistant profiles than samples on diagnosis of the disease.	[36]
Rho-123 assay and Pgp expression	62	Samples of patients in different CML phases, before or after receiving treatment, were analyzed. Pgp activity was present in 80% and Pgp expression in 84% of samples. The MDR phenotype was independent of the CML phase	[37]
Rho-123 assay and Pgp expression	245	Pgp expression and activity were present in all CML phases. The blast phase had the highest activity levels compared to chronic phase.	[38]
Rho-123 + CSA assay PhA + FTC assay	55	IM-resistant samples exhibited higher Pgp activity levels than the IM-sensitive ones. Greater Pgp activity (65.95%) was detected compared to BCRP activity (41.81%). There was no difference among the CML phases in relation to the frequency of Pgp positivity.	[39]
Rho-123 assay and Pgp expression	224	Pgp expression, but not Rho-123 efflux assay, showed differences between responder and non-responder IM-treated chronic phase patients.	[41]
Rho-123 assay and Pgp expression	38	Seventeen patients (44.7%) presented higher median Pgp expression levels in the advanced phased compared to the chronic phase. Pgp activity was found in 47.4% patients but this was not related to the CML phase.	[42]
*ABCB1* mRNA levels	30	In samples analyzed on diagnosis of CML, there was no difference between *ABCB1* mRNA levels and response to IM after 6 and 12 months of treatment.	[43]
*ABCB1* mRNA levels	63	There was no difference between *ABCB1* mRNA levels and response to IM treatment in patients in the chronic phase of CML.	[44]
*ABCB1* mRNA levels	68	In most samples on diagnosis of CML (84%), low *ABCB1* mRNA levels correlated with MMR, and high mRNA levels correlated with non-responders after IM treatment	[45]
*ABCB1* mRNA levels	83	In the chronic phase of CML, high *ABCB1* gene expression levels at the time of IM resistance were indicative of a favorable response to subsequent nilotinib treatment	[46]
*ABCB1* mRNA levels	155	Patients in the chronic phase with increased levels of *ABCB1* mRNA showed lower MMR rates at 12 and 24 months after IM treatment.	[47]

CML = chronic myeloid leukemia; MMR = major molecular response; EMR = early molecular response; IM = imatinib; Rho-123 = Rhodamine-123; Pgp = glycoprotein-P; Rho + CSA = Rhodamine-123 + Cyclosporin A; PhA + FTC = Pheophorbide + fumitremorgin C.

## References

[1] Chereda B., Melo J.V. (2015). Natural course and biology of CML. Ann. Hematol..

[2] Chen Y., Peng C., Li D., Li S. (2010). Molecular and cellular bases of chronic myeloid leukemia. Protein Cell.

[3] Cilloni D., Saglio G. (2012). Molecular pathways: BCR-ABL. Clin. Cancer Res..

[4] Druker B.J., Tamura S., Buchdunger E., Ohno S., Segal G.M., Fanning S., Zimmermann J., Lydon N.B. (1996). Effects of a selective inhibitor of the Abl tyrosine kinase on the growth of Bcr-Abl positive cells. Nat. Med..

[5] Schwetz B.A. (2001). From the Food and Drug Administration. JAMA.

[6] Apperley J.F. (2007). Part I: Mechanisms of resistance to imatinib in chronic myeloid leukaemia. Lancet Oncol..

[7] Santos F.P., Kantarjian H., Quintas-Cardama A., Cortes J. (2011). Evolution of therapies for chronic myelogenous leukemia. Cancer J..

[8] Kantarjian H., Shah N.P., Hochhaus A., Cortes J., Shah S., Ayala M., Moiraghi B., Shen Z., Mayer J., Pasquini R. (2010). Dasatinib versus imatinib in newly diagnosed chronic-phase chronic myeloid leukemia. N. Engl. J. Med..

[9] Kantarjian H.M., Shah N.P., Cortes J.E., Baccarani M., Agarwal M.B., Undurraga M.S., Wang J., Ipina J.J., Kim D.W., Ogura M. (2012). Dasatinib or imatinib in newly diagnosed chronic-phase chronic myeloid leukemia: 2-year follow-up from a randomized phase 3 trial (DASISION). Blood.

[10] Cortes J.E., Kim D.W., Pinilla-Ibarz J., le Coutre P., Paquette R., Chuah C., Nicolini F.E., Apperley J.F., Khoury H.J., Talpaz M. (2013). A phase 2 trial of ponatinib in Philadelphia chromosome-positive leukemias. N. Engl. J. Med..

[11] Kantarjian H.M., Cortes J.E., Kim D.W., Khoury H.J., Brummendorf T.H., Porkka K., Martinelli G., Durrant S., Leip E., Kelly V. (2014). Bosutinib safety and management of toxicity in leukemia patients with resistance or intolerance to imatinib and other tyrosine kinase inhibitors. Blood.

[12] Balabanov S., Braig M., Brummendorf T.H. (2014). Current aspects in resistance against tyrosine kinase inhibitors in chronic myelogenous leukemia. Drug Discov. Today Technol..

[13] Hochhaus A., Saglio G., Hughes T.P., Larson R.A., Kim D.W., Issaragrisil S., le Coutre P.D., Etienne G., Dorlhiac-Llacer P.E., Clark R.E. (2016). Long-term benefits and risks of frontline nilotinib vs imatinib for chronic myeloid leukemia in chronic phase: 5-year update of the randomized ENESTnd trial. Leukemia.

[14] Druker B.J., Guilhot F., O’Brien S.G., Gathmann I., Kantarjian H., Gattermann N., Deininger M.W., Silver R.T., Goldman J.M., Stone R.M. (2006). Five-year follow-up of patients receiving imatinib for chronic myeloid leukemia. N. Engl. J. Med..

[15] Vigil C.E., Griffiths E.A., Wang E.S., Wetzler M. (2011). Interpretation of cytogenetic and molecular results in patients treated for CML. Blood Rev..

[16] Baccarani M., Deininger M.W., Rosti G., Hochhaus A., Soverini S., Apperley J.F., Cervantes F., Clark R.E., Cortes J.E., Guilhot F. (2013). European LeukemiaNet recommendations for the management of chronic myeloid leukemia: 2013. Blood.

[17] O’Brien S.G., Guilhot F., Larson R.A., Gathmann I., Baccarani M., Cervantes F., Cornelissen J.J., Fischer T., Hochhaus A., Hughes T. (2003). Imatinib compared with interferon and low-dose cytarabine for newly diagnosed chronic-phase chronic myeloid leukemia. N. Engl. J. Med..

[18] Castagnetti F., Gugliotta G., Breccia M., Stagno F., Iurlo A., Albano F., Abruzzese E., Martino B., Levato L., Intermesoli T. (2015). Long-term outcome of chronic myeloid leukemia patients treated frontline with imatinib. Leukemia.

[19] Hughes T.P., Hochhaus A., Branford S., Muller M.C., Kaeda J.S., Foroni L., Druker B.J., Guilhot F., Larson R.A., O’Brien S.G. (2010). Long-term prognostic significance of early molecular response to imatinib in newly diagnosed chronic myeloid leukemia: An analysis from the International Randomized Study of Interferon and STI571 (IRIS). Blood.

[20] Kalmanti L., Saussele S., Lauseker M., Muller M.C., Dietz C.T., Heinrich L., Hanfstein B., Proetel U., Fabarius A., Krause S.W. (2015). Safety and efficacy of imatinib in CML over a period of 10 years: Data from the randomized CML-study IV. Leukemia.

[21] Khorashad J.S., Kelley T.W., Szankasi P., Mason C.C., Soverini S., Adrian L.T., Eide C.A., Zabriskie M.S., Lange T., Estrada J.C. (2013). BCR-ABL1 compound mutations in tyrosine kinase inhibitor-resistant CML: Frequency and clonal relationships. Blood.

[22] O’Hare T., Shakespeare W.C., Zhu X., Eide C.A., Rivera V.M., Wang F., Adrian L.T., Zhou T., Huang W.S., Xu Q. (2009). AP24534, a pan-BCR-ABL inhibitor for chronic myeloid leukemia, potently inhibits the T315I mutant and overcomes mutation-based resistance. Cancer Cell.

[23] Pagnano K.B., Bendit I., Boquimpani C., De Souza C.A., Miranda E.C., Zalcberg I., Larripa I., Nardinelli L., Silveira R.A., Fogliatto L. (2015). BCR-ABL mutations in chronic myeloid leukemia treated with tyrosine kinase inhibitors and impact on survival. Cancer Investig..

[24] Jabbour E., Kantarjian H., Jones D., Talpaz M., Bekele N., O’Brien S., Zhou X., Luthra R., Garcia-Manero G., Giles F. (2006). Frequency and clinical significance of BCR-ABL mutations in patients with chronic myeloid leukemia treated with imatinib mesylate. Leukemia.

[25] Saleh M.N., Haislip S., Sharpe J., Hess T., Gilmore J., Jackson J., Sail K.R., Ericson S.G., Chen L. (2014). Assessment of treatment and monitoring patterns and subsequent outcomes among patients with chronic myeloid leukemia treated with imatinib in a community setting. Curr. Med. Res. Opin..

[26] Nestal de Moraes G., Souza P.S., Costas F.C., Vasconcelos F.C., Reis F.R., Maia R.C. (2012). The Interface between BCR-ABL-Dependent and -Independent Resistance Signaling Pathways in Chronic Myeloid Leukemia. Leuk. Res. Treat..

[27] Copland M., Hamilton A., Elrick L.J., Baird J.W., Allan E.K., Jordanides N., Barow M., Mountford J.C., Holyoake T.L. (2006). Dasatinib (BMS-354825) targets an earlier progenitor population than imatinib in primary CML but does not eliminate the quiescent fraction. Blood.

[28] Corbin A.S., Agarwal A., Loriaux M., Cortes J., Deininger M.W., Druker B.J. (2011). Human chronic myeloid leukemia stem cells are insensitive to imatinib despite inhibition of BCR-ABL activity. J. Clin. Investig..

[29] Breier A., Gibalova L., Seres M., Barancik M., Sulova Z. (2013). New insight into p-glycoprotein as a drug target. Anticancer Agents Med. Chem..

[30] Gottesman M.M., Lavi O., Hall M.D., Gillet J.P. (2016). Toward a Better Understanding of the Complexity of Cancer Drug Resistance. Ann. Rev. Pharmacol. Toxicol..

[31] Lage H. (2008). An overview of cancer multidrug resistance: A still unsolved problem. Cell. Mol. life Sci..

[32] Ross D.D., Doyle L.A. (2004). Mining our ABCs: Pharmacogenomic approach for evaluating transporter function in cancer drug resistance. Cancer Cell.

[33] Juliano R.L., Ling V. (1976). A surface glycoprotein modulating drug permeability in Chinese hamster ovary cell mutants. Biochim. Biophys..

[34] Klukovits A., Krajcsi P. (2015). Mechanisms and therapeutic potential of inhibiting drug efflux transporters. Expert Opin. Drug Metab. Toxicol..

[35] Johnstone R.W., Ruefli A.A., Tainton K.M., Smyth M.J. (2000). A role for P-glycoprotein in regulating cell death. Leuk. Lymphoma.

[36] Carter A., Dann E.J., Katz T., Shechter Y., Oliven A., Regev R., Eytan E., Rowe J.M., Eytan G.D. (2001). Cells from chronic myelogenous leukaemia patients at presentation exhibit multidrug resistance not mediated by either MDR1 or MRP1. Br. J. Haematol..

[37] Vasconcelos F.C., Cavalcanti G.B., Silva K.L., de Meis E., Kwee J.K., Rumjanek V.M., Maia R.C. (2007). Contrasting features of MDR phenotype in leukemias by using two fluorochromes: Implications for clinical practice. Leuk. Res..

[38] Vasconcelos F.C., Silva K.L., Souza P.S., Silva L.F., Moellmann-Coelho A., Klumb C.E., Maia R.C. (2011). Variation of MDR proteins expression and activity levels according to clinical status and evolution of CML patients. Cytom. Part B Clin. Cytom..

[39] Vasconcelos F.C., Nestal de Moraes G., Moellmann-Coelho A., Maia R.C. (2013). Phosphorylated Crkl reduction levels are associated with the lowest P-glycoprotein activity levels in cells from chronic myeloid leukemia patients. Leuk. Res..

[40] Vivona D., Lima L.T., Rodrigues A.C., Bueno C.T., Alcantara G.K., Barros L.S., De Moraes Hungria V.T., Chiattone C.S., De Lourdes Lopes Ferrari Chauffaille M., Guerra-Shinohara E.M. (2014). ABCB1 haplotypes are associated with P-gp activity and affect a major molecular response in chronic myeloid leukemia patients treated with a standard dose of imatinib. Oncol. Lett..

[41] Park S.H., Park C.J., Kim D.Y., Lee B.R., Kim Y.J., Cho Y.U., Jang S. (2015). MRP1 and P-glycoprotein expression assays would be useful in the additional detection of treatment non-responders in CML patients without ABL1 mutation. Leuk. Res..

[42] Silva K.L., de Souza P.S., Nestal de Moraes G., Moellmann-Coelho A., Vasconcelos Fda C., Maia R.C. (2013). XIAP and P-glycoprotein co-expression is related to imatinib resistance in chronic myeloid leukemia cells. Leuk. Res..

[43] Razga F., Racil Z., Machova Polakova K., Buresova L., Klamova H., Zackova D., Dvorakova D., Polivkova V., Cetkovsky P., Mayer J. (2011). The predictive value of human organic cation transporter 1 and ABCB1 expression levels in different cell populations of patients with de novo chronic myelogenous leukemia. Int. J. Hematol..

[44] Malhotra H., Sharma P., Malhotra B., Bhargava S., Jasuja S., Kumar M. (2015). Molecular response to imatinib & its correlation with mRNA expression levels of imatinib influx & efflux transporters in patients with chronic myeloid leukaemia in chronic phase. Indian J. Med. Res..

[45] Da Cunha Vasconcelos F., Mauricio Scheiner M.A., Moellman-Coelho A., Mencalha A.L., Renault I.Z., Rumjanek V.M., Maia R.C. (2016). Low ABCB1 and high OCT1 levels play a favorable role in the molecular response to imatinib in CML patients in the community clinical practice. Leuk. Res..

[46] Agrawal M., Hanfstein B., Erben P., Wolf D., Ernst T., Fabarius A., Saussele S., Purkayastha D., Woodman R.C., Hofmann W.K. (2014). MDR1 expression predicts outcome of Ph+ chronic phase CML patients on second-line nilotinib therapy after imatinib failure. Leukemia.

[47] Eadie L.N., Dang P., Saunders V.A., Yeung D.T., Osborn M.P., Grigg A.P., Hughes T.P., White D.L. (2017). The clinical significance of ABCB1 overexpression in predicting outcome of CML patients undergoing first-line imatinib treatment. Leukemia.

[48] Stromskaya T.P., Rybalkina E.Y., Kruglov S.S., Zabotina T.N., Mechetner E.B., Turkina A.G., Stavrovskaya A.A. (2008). Role of P-glycoprotein in evolution of populations of chronic myeloid leukemia cells treated with imatinib. Biochem. Biokhimiia.

[49] Yeung D.T., Osborn M.P., White D.L., Branford S., Braley J., Herschtal A., Kornhauser M., Issa S., Hiwase D.K., Hertzberg M. (2015). TIDEL-II: First-line use of imatinib in CML with early switch to nilotinib for failure to achieve time-dependent molecular targets. Blood.

[50] Mahon F.X., Hayette S., Lagarde V., Belloc F., Turcq B., Nicolini F., Belanger C., Manley P.W., Leroy C., Etienne G. (2008). Evidence that resistance to nilotinib may be due to BCR-ABL, Pgp, or Src kinase overexpression. Cancer Res..

[51] Shukla S., Sauna Z.E., Ambudkar S.V. (2008). Evidence for the interaction of imatinib at the transport-substrate site(s) of the multidrug-resistance-linked ABC drug transporters ABCB1 (P-glycoprotein) and ABCG2. Leukemia.

[52] Fung K.L., Gottesman M.M. (2009). A synonymous polymorphism in a common MDR1 (ABCB1) haplotype shapes protein function. Biochim. Biophys..

[53] Vivona D., Bueno C.T., Lima L.T., Hirata R.D., Hirata M.H., Luchessi A.D., Zanichelli M.A., Chiattone C.S., Guerra-Shinohara E.M. (2012). ABCB1 haplotype is associated with major molecular response in chronic myeloid leukemia patients treated with standard-dose of imatinib. Blood Cells Mol. Dis..

[54] Dulucq S., Bouchet S., Turcq B., Lippert E., Etienne G., Reiffers J., Molimard M., Krajinovic M., Mahon F.X. (2008). Multidrug resistance gene (MDR1) polymorphisms are associated with major molecular responses to standard-dose imatinib in chronic myeloid leukemia. Blood.

[55] Wong M., Evans S., Rivory L.P., Hoskins J.M., Mann G.J., Farlow D., Clarke C.L., Balleine R.L., Gurney H. (2005). Hepatic technetium Tc 99m-labeled sestamibi elimination rate and ABCB1 (MDR1) genotype as indicators of ABCB1 (P-glycoprotein) activity in patients with cancer. Clin. Pharmacol. Ther..

[56] Hamada A., Miyano H., Watanabe H., Saito H. (2003). Interaction of imatinib mesilate with human P-glycoprotein. J. Pharmaco. Exp. Ther..

[57] Dulucq S., Krajinovic M. (2010). The pharmacogenetics of imanitib. Genome Med..

[58] Ni L.N., Li J.Y., Miao K.R., Qiao C., Zhang S.J., Qiu H.R., Qian S.X. (2011). Multidrug resistance gene (MDR1) polymorphisms correlate with imatinib response in chronic myeloid leukemia. Med. Oncol..

[59] Deenik W., van der Holt B., Janssen J.J., Chu I.W., Valk P.J., Ossenkoppele G.J., van der Heiden I.P., Sonneveld P., van Schaik R.H., Cornelissen J.J. (2010). Polymorphisms in the multidrug resistance gene MDR1 (ABCB1) predict for molecular resistance in patients with newly diagnosed chronic myeloid leukemia receiving high-dose imatinib. Blood.

[60] Au A., Aziz Baba A., Goh A.S., Wahid Fadilah S.A., Teh A., Rosline H., Ankathil R. (2014). Association of genotypes and haplotypes of multi-drug transporter genes ABCB1 and ABCG2 with clinical response to imatinib mesylate in chronic myeloid leukemia patients. Biomed. Pharmacother..

[61] Adeagbo B.A., Bolaji O.O., Olugbade T.A., Durosinmi M.A., Bolarinwa R.A., Masimirembwa C. (2016). Influence of CYP3A5*3 and ABCB1 C3435T on clinical outcomes and trough plasma concentrations of imatinib in Nigerians with chronic myeloid leukaemia. J. Clin. Pharm. Ther..

[62] Maffioli M., Camos M., Gaya A., Hernandez-Boluda J.C., Alvarez-Larran A., Domingo A., Granell M., Guillem V., Vallansot R., Costa D. (2011). Correlation between genetic polymorphisms of the hOCT1 and MDR1 genes and the response to imatinib in patients newly diagnosed with chronic-phase chronic myeloid leukemia. Leuk. Res..

[63] Angelini S., Soverini S., Ravegnini G., Barnett M., Turrini E., Thornquist M., Pane F., Hughes T.P., White D.L., Radich J. (2013). Association between imatinib transporters and metabolizing enzymes genotype and response in newly diagnosed chronic myeloid leukemia patients receiving imatinib therapy. Haematologica.

[64] Ali M.A., Elsalakawy W.A. (2014). ABCB1 haplotypes but not individual SNPs predict for optimal response/failure in Egyptian patients with chronic-phase chronic myeloid leukemia receiving imatinib mesylate. Med. Oncol..

[65] Elghannam D.M., Ibrahim L., Ebrahim M.A., Azmy E., Hakem H. (2014). Association of MDR1 gene polymorphism (G2677T) with imatinib response in Egyptian chronic myeloid leukemia patients. Hematology.

[66] Kim D.H., Sriharsha L., Xu W., Kamel-Reid S., Liu X., Siminovitch K., Messner H.A., Lipton J.H. (2009). Clinical relevance of a pharmacogenetic approach using multiple candidate genes to predict response and resistance to imatinib therapy in chronic myeloid leukemia. Clin. Cancer Res..

[67] Takahashi N., Miura M., Scott S.A., Kagaya H., Kameoka Y., Tagawa H., Saitoh H., Fujishima N., Yoshioka T., Hirokawa M. (2010). Influence of CYP3A5 and drug transporter polymorphisms on imatinib trough concentration and clinical response among patients with chronic phase chronic myeloid leukemia. J. Hum. Genet..

[68] Vine J., Cohen S.B., Ruchlemer R., Goldschmidt N., Levin M., Libster D., Gural A., Gatt M.E., Lavie D., Ben-Yehuda D. (2014). Polymorphisms in the human organic cation transporter and the multidrug resistance gene: Correlation with imatinib levels and clinical course in patients with chronic myeloid leukemia. Leuk. Lymphoma.

[69] Kimchi-Sarfaty C., Oh J.M., Kim I.W., Sauna Z.E., Calcagno A.M., Ambudkar S.V., Gottesman M.M. (2007). A “silent” polymorphism in the MDR1 gene changes substrate specificity. Science.

[70] Hoffmeyer S., Burk O., von Richter O., Arnold H.P., Brockmoller J., Johne A., Cascorbi I., Gerloff T., Roots I., Eichelbaum M. (2000). Functional polymorphisms of the human multidrug-resistance gene: Multiple sequence variations and correlation of one allele with P-glycoprotein expression and activity in vivo. Proc. Natl. Acad. Sci. USA.

[71] Song P., Lamba J.K., Zhang L., Schuetz E., Shukla N., Meibohm B., Yates C.R. (2006). G2677T and C3435T genotype and haplotype are associated with hepatic ABCB1 (MDR1) expression. J. Clin. Pharmacol..

[72] Penna G., Allegra A., Alonci A., Aguennouz M., Garufi A., Cannavo A., Gerace D., Alibrandi A., Musolino C. (2011). MDR-1 polymorphisms (G2677T and C3435T) in B-chronic lymphocytic leukemia: An impact on susceptibility and prognosis. Med. Oncol..

[73] Goreva O.B., Grishanova A.Y., Mukhin O.V., Domnikova N.P., Lyakhovich V.V. (2003). Possible prediction of the efficiency of chemotherapy in patients with lymphoproliferative diseases based on MDR1 gene G2677T and C3435T polymorphisms. Bull. Exp. Biol. Med..

[74] Jamroziak K., Mlynarski W., Balcerczak E., Mistygacz M., Trelinska J., Mirowski M., Bodalski J., Robak T. (2004). Functional C3435T polymorphism of MDR1 gene: An impact on genetic susceptibility and clinical outcome of childhood acute lymphoblastic leukemia. Eur. J. Haematol..

[75] Yaya K., Hind D., Meryem Q., Asma Q., Said B., Sellama N. (2014). Single nucleotide polymorphisms of multidrug resistance gene 1 (MDR1) and risk of chronic myeloid leukemia. Tumour Biol..

[76] Gurney H., Wong M., Balleine R.L., Rivory L.P., McLachlan A.J., Hoskins J.M., Wilcken N., Clarke C.L., Mann G.J., Collins M. (2007). Imatinib disposition and ABCB1 (MDR1, P-glycoprotein) genotype. Clin. Pharmacol. Ther..

[77] Galeotti L., Ceccherini F., Domingo D., Laurino M., Polillo M., Di Paolo A., Barate C., Fava C., D’Avolio A., Cervetti G. (2017). Association of the hOCT1/ABCB1 genotype with efficacy and tolerability of imatinib in patients affected by chronic myeloid leukemia. Cancer Chemother. Pharmacol..

[78] Ambudkar S.V., Sauna Z.E., Gottesman M.M., Szakacs G. (2005). A novel way to spread drug resistance in tumor cells: Functional intercellular transfer of P-glycoprotein (ABCB1). Trends Pharmacol. Sci..

[79] Bebawy M., Combes V., Lee E., Jaiswal R., Gong J., Bonhoure A., Grau G.E. (2009). Membrane microparticles mediate transfer of P-glycoprotein to drug sensitive cancer cells. Leukemia.

[80] Souza P.S., Madigan J.P., Gillet J.P., Kapoor K., Ambudkar S.V., Maia R.C., Gottesman M.M., Fung K.L. (2015). Expression of the multidrug transporter P-glycoprotein is inversely related to that of apoptosis-associated endogenous TRAIL. Exp. Cell Res..

[81] McDermott M., Eustace A.J., Busschots S., Breen L., Crown J., Clynes M., O’Donovan N., Stordal B. (2014). In vitro Development of Chemotherapy and Targeted Therapy Drug-Resistant Cancer Cell Lines: A Practical Guide with Case Studies. Front. Oncol..

[82] Rumjanek V.M., Trindade G.S., Wagner-Souza K., Meletti-de-Oliveira M.C., Marques-Santos L.F., Maia R.C., Capella M.A.M. (2001). Multidrug resistance in tumour cells: Characterisation of the multidrug resistant cell line K562-Lucena 1. An. Acad. Bras. Cienc..

[83] Daflon-Yunes N., Pinto-Silva F.E., Vidal R.S., Novis B.F., Berguetti T., Lopes R.R., Polycarpo C., Rumjanek V.M. (2013). Characterization of a multidrug-resistant chronic myeloid leukemia cell line presenting multiple resistance mechanisms. Mol. Cell. Biochem..

[84] Moreira M.A., Bagni C., de Pinho M.B., Mac-Cormick T.M., dos Santos Mota M., Pinto-Silva F.E., Daflon-Yunes N., Rumjanek V.M. (2014). Changes in gene expression profile in two multidrug resistant cell lines derived from a same drug sensitive cell line. Leuk. Res..

[85] Mahon F.X., Deininger M.W., Schultheis B., Chabrol J., Reiffers J., Goldman J.M., Melo J.V. (2000). Selection and characterization of BCR-ABL positive cell lines with differential sensitivity to the tyrosine kinase inhibitor STI571: Diverse mechanisms of resistance. Blood.

[86] Alves R., Fonseca A.R., Goncalves A.C., Ferreira-Teixeira M., Lima J., Abrantes A.M., Alves V., Rodrigues-Santos P., Jorge L., Matoso E. (2015). Drug transporters play a key role in the complex process of Imatinib resistance in vitro. Leuk. Res..

[87] Tiwari A.K., Sodani K., Wang S.R., Kuang Y.H., Ashby C.R., Chen X., Chen Z.S. (2009). Nilotinib (AMN107, Tasigna) reverses multidrug resistance by inhibiting the activity of the ABCB1/Pgp and ABCG2/BCRP/MXR transporters. Biochem. Pharmacol..

[88] Hiwase D.K., Saunders V., Hewett D., Frede A., Zrim S., Dang P., Eadie L., To L.B., Melo J., Kumar S. (2008). Dasatinib cellular uptake and efflux in chronic myeloid leukemia cells: Therapeutic implications. Clin. Cancer Res..

[89] Redaelli S., Perini P., Ceccon M., Piazza R., Rigolio R., Mauri M., Boschelli F., Giannoudis A., Gambacorti-Passerini C. (2015). In vitro and in vivo identification of ABCB1 as an efflux transporter of bosutinib. J. Hematol. Oncol..

[90] Peng X.X., Tiwari A.K., Wu H.C., Chen Z.S. (2012). Overexpression of P-glycoprotein induces acquired resistance to imatinib in chronic myelogenous leukemia cells. Chin. J. Cancer.

[91] Hiwase D.K., White D., Zrim S., Saunders V., Melo J.V., Hughes T.P. (2010). Nilotinib-mediated inhibition of ABCB1 increases intracellular concentration of dasatinib in CML cells: Implications for combination TKI therapy. Leukemia.

[92] Dohse M., Scharenberg C., Shukla S., Robey R.W., Volkmann T., Deeken J.F., Brendel C., Ambudkar S.V., Neubauer A., Bates S.E. (2010). Comparison of ATP-binding cassette transporter interactions with the tyrosine kinase inhibitors imatinib, nilotinib, and dasatinib. Drug Metab. Dispos..

[93] Hu X.F., Slater A., Kantharidis P., Rischin D., Juneja S., Rossi R., Lee G., Parkin J.D., Zalcberg J.R. (1999). Altered multidrug resistance phenotype caused by anthracycline analogues and cytosine arabinoside in myeloid leukemia. Blood.

[94] Callaghan R., Crowley E., Potter S., Kerr I.D. (2008). P-glycoprotein: So many ways to turn it on. J. Clin. Pharmacol..

[95] De Souza P.S., Faccion R.S., Bernardo P.S., Maia R.C. (2016). Membrane microparticles: Shedding new light into cancer cell communication. J. Cancer Res. Clin. Oncol..

[96] Jaiswal R., Gong J., Sambasivam S., Combes V., Mathys J.M., Davey R., Grau G.E., Bebawy M. (2012). Microparticle-associated nucleic acids mediate trait dominance in cancer. FASEB J..

[97] Lu J.F., Luk F., Gong J., Jaiswal R., Grau G.E., Bebawy M. (2013). Microparticles mediate MRP1 intercellular transfer and the re-templating of intrinsic resistance pathways. Pharmacol. Res..

[98] Lopes-Rodrigues V., Di Luca A., Sousa D., Seca H., Meleady P., Henry M., Lima R.T., O’Connor R., Vasconcelos M.H. (2016). Multidrug resistant tumour cells shed more microvesicle-like EVs and less exosomes than their drug-sensitive counterpart cells. Biochim. Biophys. Acta.

[99] Milani G., Lana T., Bresolin S., Aveic S., Pasto A., Frasson C., Te Kronnie G. (2017). Expression Profiling of Circulating Microvesicles Reveals Intercellular Transmission of Oncogenic Pathways. Mol. Cancer Res..

[100] Corrado C., Raimondo S., Saieva L., Flugy A.M., De Leo G., Alessandro R. (2014). Exosome-mediated crosstalk between chronic myelogenous leukemia cells and human bone marrow stromal cells triggers an interleukin 8-dependent survival of leukemia cells. Cancer Lett..

[101] Raimondo S., Saieva L., Corrado C., Fontana S., Flugy A., Rizzo A., De Leo G., Alessandro R. (2015). Chronic myeloid leukemia-derived exosomes promote tumor growth through an autocrine mechanism. Cell Commun. Signal..

[102] Corrado C., Saieva L., Raimondo S., Santoro A., De Leo G., Alessandro R. (2016). Chronic myelogenous leukaemia exosomes modulate bone marrow microenvironment through activation of epidermal growth factor receptor. J. Cell. Mol. Med..

[103] Caivano A., Laurenzana I., De Luca L., La Rocca F., Simeon V., Trino S., D’Auria F., Traficante A., Maietti M., Izzo T. (2015). High serum levels of extracellular vesicles expressing malignancy-related markers are released in patients with various types of hematological neoplastic disorders. Tumour Biol..

[104] Li Z., Hu S., Wang J., Cai J., Xiao L., Yu L., Wang Z. (2010). MiR-27a modulates MDR1/P-glycoprotein expression by targeting HIPK2 in human ovarian cancer cells. Gynecol. Oncol..

[105] Li H., Yang B.B. (2013). Friend or foe: The role of microRNA in chemotherapy resistance. Acta Pharmacol. Sin..

[106] Vasudevan S., Tong Y., Steitz J.A. (2007). Switching from repression to activation: MicroRNAs can up-regulate translation. Science.

[107] Haenisch S., Werk A.N., Cascorbi I. (2014). MicroRNAs and their relevance to ABC transporters. Br. J. Clin. Pharmacol..

[108] Lopes-Rodrigues V., Seca H., Sousa D., Sousa E., Lima R.T., Vasconcelos M.H. (2014). The network of P-glycoprotein and microRNAs interactions. Int. J. Cancer.

[109] Zhu H., Wu H., Liu X., Evans B.R., Medina D.J., Liu C.G., Yang J.M. (2008). Role of MicroRNA miR-27a and miR-451 in the regulation of MDR1/P-glycoprotein expression in human cancer cells. Biochem. Pharmacol..

[110] Toscano-Garibay J.D., Aquino-Jarquin G. (2012). Regulation exerted by miRNAs in the promoter and UTR sequences: MDR1/P-gp expression as a particular case. DNA Cell Biol..

[111] Feng D.D., Zhang H., Zhang P., Zheng Y.S., Zhang X.J., Han B.W., Luo X.Q., Xu L., Zhou H., Qu L.H. (2011). Down-regulated miR-331-5p and miR-27a are associated with chemotherapy resistance and relapse in leukaemia. J. Cell. Mol. Med..

[112] Fei J., Li Y., Zhu X., Luo X. (2012). miR-181a post-transcriptionally downregulates oncogenic RalA and contributes to growth inhibition and apoptosis in chronic myelogenous leukemia (CML). PLoS ONE.

[113] Mosakhani N., Mustjoki S., Knuutila S. (2013). Down-regulation of miR-181c in imatinib-resistant chronic myeloid leukemia. Mol. Cytogenet..

[114] Fallah P., Amirizadeh N., Poopak B., Toogeh G., Arefian E., Kohram F., Hosseini Rad S.M., Kohram M., Teimori Naghadeh H., Soleimani M. (2015). Expression pattern of key microRNAs in patients with newly diagnosed chronic myeloid leukemia in chronic phase. Int. J. Lab. Hematol..

[115] Flamant S., Ritchie W., Guilhot J., Holst J., Bonnet M.L., Chomel J.C., Guilhot F., Turhan A.G., Rasko J.E. (2010). Micro-RNA response to imatinib mesylate in patients with chronic myeloid leukemia. Haematologica.

[116] Li Y., Zhao L., Li N., Miao Y., Zhou H., Jia L. (2017). miR-9 regulates the multidrug resistance of chronic myelogenous leukemia by targeting ABCB1. Oncol. Rep..

[117] Ohyashiki K., Umezu T., Katagiri S., Kobayashi C., Azuma K., Tauchi T., Okabe S., Fukuoka Y., Ohyashiki J.H. (2016). Downregulation of Plasma miR-215 in Chronic Myeloid Leukemia Patients with Successful Discontinuation of Imatinib. Int. J. Mol. Sci..

[118] Yap E., Norziha Z.A., Simbun A., Tumian N.R., Cheong S.K., Leong C.F., Wong C.L. (2017). MicroRNAs that affect the Fanconi Anemia/BRCA pathway are downregulated in imatinib-resistant chronic myeloid leukemia patients without detectable BCR-ABL kinase domain mutations. Leuk. Res..

[119] Nestal de Moraes G., Silva K.L., Vasconcelos F.C., Maia R.C. (2011). Survivin overexpression correlates with an apoptosis-resistant phenotype in chronic myeloid leukemia cells. Oncol. Rep..

[120] Reis F.R., Vasconcelos F.C., Pereira D.L., Moellman-Coelho A., Silva K.L., Maia R.C. (2011). Survivin and P-glycoprotein are associated and highly expressed in late phase chronic myeloid leukemia. Oncol. Rep..

[121] Souza P.S., Vasconcelos F.C., De Souza Reis F.R., Nestal De Moraes G., Maia R.C. (2011). P-glycoprotein and survivin simultaneously regulate vincristine-induced apoptosis in chronic myeloid leukemia cells. Int. J. Oncol..

[122] Bernardo P.S., Reis F.R., Maia R.C. (2012). Imatinib increases apoptosis index through modulation of survivin subcellular localization in the blast phase of CML cells. Leuk. Res..

[123] Stenner M., Weinell A., Ponert T., Hardt A., Hahn M., Preuss S.F., Guntinas-Lichius O., Klussmann J.P. (2010). Cytoplasmic expression of survivin is an independent predictor of poor prognosis in patients with salivary gland cancer. Histopathology.

[124] Sokal J.E., Cox E.B., Baccarani M., Tura S., Gomez G.A., Robertson J.E., Tso C.Y., Braun T.J., Clarkson B.D., Cervantes F. (1984). Prognostic discrimination in “good-risk” chronic granulocytic leukemia. Blood.

[125] Seca H., Lima R.T., Guimaraes J.E., Helena Vasconcelos M. (2011). Simultaneous targeting of P-gp and XIAP with siRNAs increases sensitivity of P-gp overexpressing CML cells to imatinib. Hematology.

[126] List A.F., Kopecky K.J., Willman C.L., Head D.R., Slovak M.L., Douer D., Dakhil S.R., Appelbaum F.R. (2002). Cyclosporine inhibition of P-glycoprotein in chronic myeloid leukemia blast phase. Blood.

[127] Maia R.C., Carrico M.K., Klumb C.E., Noronha H., Coelho A.M., Vasconcelos F.C., Ruimanek V.M. (1997). Clinical approach to circumvention of multidrug resistance in refractory leukemic patients: Association of cyclosporin A with etoposide. J. Exp. Clin. Cancer Res..

[128] Illmer T., Schaich M., Platzbecker U., Freiberg-Richter J., Oelschlagel U., von Bonin M., Pursche S., Bergemann T., Ehninger G., Schleyer E. (2004). P-glycoprotein-mediated drug efflux is a resistance mechanism of chronic myelogenous leukemia cells to treatment with imatinib mesylate. Leukemia.

[129] Mahon F.X., Belloc F., Lagarde V., Chollet C., Moreau-Gaudry F., Reiffers J., Goldman J.M., Melo J.V. (2003). MDR1 gene overexpression confers resistance to imatinib mesylate in leukemia cell line models. Blood.

[130] Thomas J., Wang L., Clark R.E., Pirmohamed M. (2004). Active transport of imatinib into and out of cells: Implications for drug resistance. Blood.

[131] Raaijmakers M.H. (2007). ATP-binding-cassette transporters in hematopoietic stem cells and their utility as therapeutical targets in acute and chronic myeloid leukemia. Leukemia.

[132] Kim M., Turnquist H., Jackson J., Sgagias M., Yan Y., Gong M., Dean M., Sharp J.G., Cowan K. (2002). The multidrug resistance transporter ABCG2 (breast cancer resistance protein 1) effluxes Hoechst 33342 and is overexpressed in hematopoietic stem cells. Clin. Cancer Res..

[133] Wang F., Wang X.K., Shi C.J., Zhang H., Hu Y.P., Chen Y.F., Fu L.W. (2014). Nilotinib enhances the efficacy of conventional chemotherapeutic drugs in CD34(+)CD38(−) stem cells and ABC transporter overexpressing leukemia cells. Molecules.

[134] Sen R., Natarajan K., Bhullar J., Shukla S., Fang H.B., Cai L., Chen Z.S., Ambudkar S.V., Baer M.R. (2012). The novel BCR-ABL and FLT3 inhibitor ponatinib is a potent inhibitor of the MDR-associated ATP-binding cassette transporter ABCG2. Mol. Cancer Ther..

[135] Lu L., Saunders V.A., Leclercq T.M., Hughes T.P., White D.L. (2015). Ponatinib is not transported by ABCB1, ABCG2 or OCT-1 in CML cells. Leukemia.

[136] Rai D., Singh J.K., Roy N., Panda D. (2008). Curcumin inhibits FtsZ assembly: An attractive mechanism for its antibacterial activity. Biochem. J..

[137] Lin S.S., Huang H.P., Yang J.S., Wu J.Y., Hsia T.C., Lin C.C., Lin C.W., Kuo C.L., Gibson Wood W., Chung J.G. (2008). DNA damage and endoplasmic reticulum stress mediated curcumin-induced cell cycle arrest and apoptosis in human lung carcinoma A-549 cells through the activation caspases cascade- and mitochondrial-dependent pathway. Cancer Lett..

[138] Lopes-Rodrigues V., Oliveira A., Correia-da-Silva M., Pinto M., Lima R.T., Sousa E., Vasconcelos M.H. (2017). A novel curcumin derivative which inhibits P-glycoprotein, arrests cell cycle and induces apoptosis in multidrug resistance cells. Bioorg. Med. Chem..

[139] Mapoung S., Pitchakarn P., Yodkeeree S., Ovatlarnporn C., Sakorn N., Limtrakul P. (2016). Chemosensitizing effects of synthetic curcumin analogs on human multi-drug resistance leukemic cells. Chem. Biol. Interact..

[140] Zhang J.Y., Lin M.T., Yi T., Tang Y.N., Fan L.L., He X.C., Zhao Z.Z., Chen H.B. (2013). Apoptosis sensitization by Euphorbia factor L1 in ABCB1-mediated multidrug resistant K562/ADR cells. Molecules.

[141] Netto C.D., da Silva A.J., Salustiano E.J., Bacelar T.S., Rica I.G., Cavalcante M.C., Rumjanek V.M., Costa P.R. (2010). New pterocarpanquinones: Synthesis, antineoplasic activity on cultured human malignant cell lines and TNF-alpha modulation in human PBMC cells. Bioorg. Med. Chem..

[142] Maia R.C., Vasconcelos F.C., de Sa Bacelar T., Salustiano E.J., da Silva L.F., Pereira D.L., Moellman-Coelho A., Netto C.D., da Silva A.J., Rumjanek V.M. (2011). LQB-118, a pterocarpanquinone structurally related to lapachol [2-hydroxy-3-(3-methyl-2-butenyl)-1,4-naphthoquinone]: A novel class of agent with high apoptotic effect in chronic myeloid leukemia cells. Investig. New Drugs.

[143] De Sa Bacelar T., da Silva A.J., Costa P.R., Rumjanek V.M. (2013). The pterocarpanquinone LQB 118 induces apoptosis in tumor cells through the intrinsic pathway and the endoplasmic reticulum stress pathway. Anticancer Drugs.

[144] De Faria F.C., Leal M.E., Bernardo P.S., Costa P.R., Maia R.C. (2015). NFkappaB pathway and microRNA-9 and -21 are involved in sensitivity to the pterocarpanquinone LQB-118 in different CML cell lines. Anticancer Agents Med. Chem..

